# Low Doses of Gamma-Irradiation Induce an Early Bystander Effect in Zebrafish Cells Which Is Sufficient to Radioprotect Cells

**DOI:** 10.1371/journal.pone.0092974

**Published:** 2014-03-25

**Authors:** Sandrine Pereira, Véronique Malard, Jean-Luc Ravanat, Anne-Hélène Davin, Jean Armengaud, Nicolas Foray, Christelle Adam-Guillermin

**Affiliations:** 1 Institut de Radioprotection et de Sûreté Nucléaire, PRP-Environnement/SERIS, Laboratoire d’Ecotoxicologie des Radionucléides, Cadarache, St Paul Lez Durance, France; 2 CRCL - UMR INSERM 1052 - CNRS 5286, Equipe de Radiobiologie, Cheney A- 1éme étage, Lyon, France; 3 CEA, DSV, IBEB, Lab Biochim System Perturb, Bagnols-sur-Cèze, France; 4 Laboratoire des Lésions des Acides Nucléiques, INAC/Scib UMR E3 CEA-UJF, CEA Grenoble, Grenoble, France; German Cancer Research Center, Germany

## Abstract

The term “bystander effect” is used to describe an effect in which cells that have not been exposed to radiation are affected by irradiated cells though various intracellular signaling mechanisms. In this study we analyzed the kinetics and mechanisms of bystander effect and radioadaptation in embryonic zebrafish cells (ZF4) exposed to chronic low dose of gamma rays. ZF4 cells were irradiated for 4 hours with total doses of gamma irradiation ranging from 0.01–0.1 Gy. In two experimental conditions, the transfer of irradiated cells or culture medium from irradiated cells results in the occurrence of DNA double strand breaks in non-irradiated cells (assessed by the number of γ-H2AX foci) that are repaired at 24 hours post-irradiation whatever the dose. At low total irradiation doses the bystander effect observed does not affect DNA repair mechanisms in targeted and bystander cells. An increase in global methylation of ZF4 cells was observed in irradiated cells and bystander cells compared to control cells. We observed that pre-irradiated cells which are then irradiated for a second time with the same doses contained significantly less γ-H2AX foci than in 24 h gamma-irradiated control cells. We also showed that bystander cells that have been in contact with the pre-irradiated cells and then irradiated alone present less γ-H2AX foci compared to the control cells. This radioadaptation effect is significantly more pronounced at the highest doses. To determine the factors involved in the early events of the bystander effect, we performed an extensive comparative proteomic study of the ZF4 secretomes upon irradiation. In the experimental conditions assayed here, we showed that the early events of bystander effect are probably not due to the secretion of specific proteins neither the oxidation of these secreted proteins. These results suggest that early bystander effect may be due probably to a combination of multiple factors.

## Introduction

To address potential health risks associated with radiation exposure, the long-term biological consequences of targeted and non-targeted effects in exposed cells and their progeny should be described. The term bystander effect has been used to describe an effect in which cells that have not been exposed to radiation are affected by irradiated cells though various intercellular signaling mechanisms [1], [2], [3]. This phenomenon occurs when cells that are not directly exposed to radiation, but receive signals from irradiated cells, respond as though they were irradiated [4], [5]. Micronucleus formation, sister chromatid exchange, DNA double strand breaks, genomic instability are bystander effects that have been reported in non-irradiated cells and have been extensively studied. Specifically those induced by ionizing irradiation have been assessed through various approaches including transfer of conditioned medium from irradiated cells [6], [7], [8], low particle fluence irradiation, where only a few percent of cells are irradiated [9], and targeted irradiation of single cells and subcellular structures [10], [11], [12]. They have been shown to occur in both tumour and normal cell types. They have been observed for a range of end points, including cell killing [13]. The involvement of soluble factors like reactive oxygen species (ROS) [14], [15], [12], nitric oxide (NO) [11], [12], [16] and cytokines released from irradiated cells as well as gap junction intercellular communication [14], [15], [16], [17] have recently been reported. End points used for the study of bystander effects *in vitro* have included micronuclei formation [18], [10], gene mutations and genomic instability [19], gene expression changes [2], [20], transformation [21], proliferation [22], cell survival, apoptosis [24], [6], cell cycle arrest [23] and the induction of γ-H2AX foci in bystander cells [23], [16], [20], [25], [26], [27].

DNA double-strand breaks (DSBs) are considered the key lesions responsible for radiation-induced cell death because they are less easily repaired than other DNA damages. *In vitro* research has revealed that the severity of the bystander effect depends on whether the cell type producing or receiving the bystander signal is DNA repair-proficient or DNA repair-deficient [4], [28]. Although numerous studies on bystander effect have been reported, the mechanisms involved in bystander signaling have only partially been elucidated and are likely dependent on cell type and end points being investigated. Furthermore, bystander mechanisms have been proposed to be epigenetic in nature [29].

The radioadaptive response is defined as induction of radioresistance to subsequent higher doses of radiation by a priming irradiation with low radiation doses [30], [31]. This response may therefore constitute one of the protective effects of low-dose radiation. Indeed some authors have shown that radiation-induced bystander effects may play a role in radioadaptive responses [32]. Some authors have studied the benefit in terms of the induction of radioadaptive response (RAR) between zebrafish embryos by communication of such bystander signals [33], [34], [35]. RAR is a kind of low-dose radiation effect, which occurs when a small initial priming dose induces a decrease of the biological effectiveness of a subsequent large radiation dose that would normally induce a great deal of damage.

Most of studies to assess the bystander effect and the adaptive response in fish were *in vivo* studies [33]–[40] with exposure to radiation doses ranging from 0.1 to 4 Gy of either alpha, X- or gamma rays. Nevertheless, none of these studies have clearly analyzed the kinetics and mechanisms of bystander effects and radioadaptation for a chronic low dose of gamma irradiation. In particular, the temporal and spatial dependence of the transfer of bystander effects on zebrafishes has not been studied. In addition, since embryogenesis is a specific radiosensitive stage of the vertebrate life cycle zebrafish, embryos are ideal for evaluating genotoxic stress as well as radiation-related studies [41], [42].

In the present study, we analyze the radiation-induced bystander effect and the radioadaptive response in embryonic zebrafish cells (ZF4) exposed to chronic gamma rays. Based on the protocols from the literature, ZF4 cells were irradiated with gamma rays doses ranging from 0.01–0.1 Gy. Under different experimental conditions, the production and repair of DSBs in the embryonic zebrafish ZF4 cell line was assessed. In addition, epigenetics effects were assessed via global methylation analysis and a determination of the factors involved in the bystander effect and radioadaptative response was performed.

## Materials and Methods

### Cell Culture

Embryonic zebrafish fibroblasts (ZF4) were obtained from the ATCC (CRL-2050). Cells were routinely cultured at 28°C and 5% CO2 as monolayers with Glutamax Dulbecco’s modified Eagle’s minimum-Ham’s F12 medium (DMEM) (Gibco-Invitrogen), supplemented with 20% of inactivated fetal calf serum, penicillin and streptomycin. Cells for irradiation experiments were cultured without fetal calf serum. All experiments were performed with cells in plateau phase of growth (95–99% of cells in G0/G1) at passages 7, 8 and 10 to overcome any bias generated by cell cycle.

### Gamma Irradiations

For chronic irradiations, ^137^Cs sources were purchased from CERCA-LEA (Framatome ANP, Pierrelatte, France). Either a solution of ^137^Cs in a polystyrene tube (20 or 200 MBq in HCl 0.1 M) or as a solid ^137^Cs line source (1.85 GBq) was used to obtain gamma rays for a period of 24 h. Nominal dose rates of 0.7, 7, 70, 550 mGy/d were verified by TLD measurements. Cells were irradiated according to the procedure described by Gilbin et al [43].

### Bystander Experiments

**Figure 1:**
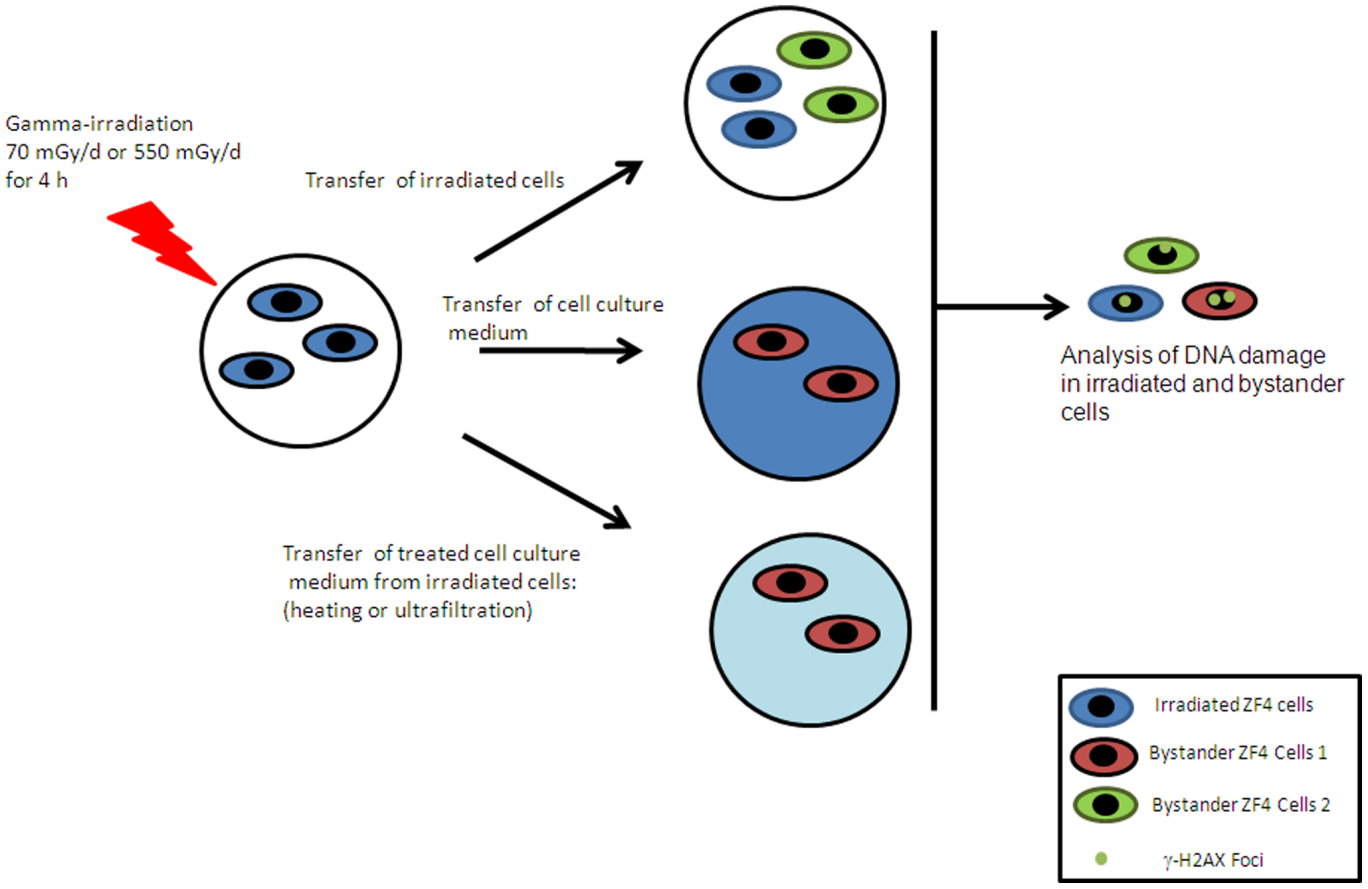
Experimental set up for bystander experiments.

One day before irradiation, 1×10^5^ cells were seeded in 18 mm diameter slides. Cells were irradiated for 4 h at 28°C with a total dose of 12 mGy (dose rate of 70 mGy/d) or 92 mGy (dose rate of 550 mGy/d) of gamma rays generated by a ^137^Cs gamma irradiator (see below). Then either slides with irradiated cells were co-cultured with non-irradiated cells (called bystander cells 2) or the medium from irradiated cells transferred to the non-irradiated cells (called bystander cells 1). Cells were co-cultured in these two conditions for 1–24 h (see Figure 1 for experimental design). Irradiated and non-irradiated cells were then fixed and analyzed for the occurrence of γ-H2AX foci at the time periods indicated (1 h, 2 h, 4 h and 24 h). To assess the nature of the secreted factor(s) involved in early bystander effect, the irradiated culture medium was either heated at 100°C for 10 min or ultrafiltered using Amicon ultracentrifugal filter devices with a 3 kDa cut-off (Millipore) operated at 4°C by centrifugation at 4500 g. The resulting samples were then applied on non-irradiated cells within 1 h. After the incubation the occurrence of γ-H2AX was observed in the treated cells.

### Radioadaptation Experiments

One day before irradiation, 1×10^5^ cells were seeded in 18 mm diameter slides. Cells were irradiated 4 h at 28°C with a total dose of 12 mGy (dose rate of 70 mGy/d) or 92 mGy (dose rate of 550 mGy/d) of gamma rays that were generated by a ^137^Cs gamma irradiator (see above). Irradiated cells were placed with non-irradiated cells and co-cultured for 1 h at 28°C and then further irradiated 20 h at 28°C with a challenging dose of 58 mGy (dose rate of 70 mGy/d) or 460 mGy (dose rate of 550 mGy/d). Irradiated and non-irradiated cells were fixed and analyzed for the occurrence of γ-H2AX foci at the time periods indicated (1 h, 2 h, 4 h and 24 h).

### Immunofluorescence

The immunofluorescence protocol employed for DNA repair and signaling factors are described elsewhere [44]. All chemicals used in immunofluorescence buffers were purchased from Sigma–Aldrich, France. Briefly, cells were fixed in 4% paraformaldehyde 2% sucrose PBS (Phosphate Buffered Saline solution) for 15 min at room temperature and permeabilized in 20 mM HEPES, pH 7.4, 50 mM NaCl, 3 mM MgCl2, 300 mM sucrose 0.5% Triton X-100 for three min. Thereafter, coverslips were washed in PBS prior to immunostaining. Anti-pH2AXser139 antibodies provided by Upstate Biotechnology-Euromedex (Mundolsheim, France) were used at 1∶800. Incubations with anti-mouse fluorescein (FITC) secondary antibodies were performed at 1∶100 at 37°C for 20 min. Slides were mounted in 4′,6′-diamidino-2-phenyl-indole (DAPI)-stained Vectashield (Abcys) and examined with a Nikon fluorescence microscope (Nikon Eclipse E600). We observed 200 to 600 nuclei per experiment. DAPI staining allowed us to indirectly evaluate yield of G1 cells (nuclei with homogeneous DAPI staining) and micronuclei. 1000 cells were counted for micronuclei analysis.

### DNA Purification and Analysis

DNA extraction from ZF4 cells (13.10^6^ cells per sample) was done with DNeasy Blood & Tissue Kit (QIAGEN, France). Samples comprising 3 to 5 μg of each DNA were analyzed for methylation by High-performance liquid chromatography (HPLC) coupled through electrospray ionization to tandem mass spectrometry (MS/MS) *vide infra.* subsequently to DNA hydrolysis.

### 5-methyl-2-deoxycytidine and 2-deoxycytidine Quantification

The 2′-deoxycytosine (dCyd) and 5-methyl-2′-deoxycytosine (5-MedCyd) compounds were detected and quantified by mass spectrometry with the so-called multiple reactions monitoring mode (mrm) using transitions 228 → 112 and 242 → 126, respectively (Meador et al, 2010). These measurements were taken with a TSQ Quantum Ultra (Thermo Fisher Scientific INC) tandem mass spectrometer. Conditions for DNA digestion and HPLC separation were similar to those described previously [45]. Quantification was performed by external calibration.

### Secretome Samples

For mass spectrometry analysis of secretomes, cell layers were thoroughly washed in serum free medium to eliminate protein contamination, then irradiated at either 70 or 550 mGy/d or left unirradiated for 4 hours in serum free medium. Culture supernatants were then collected (5.5 mL per condition, biological duplicates) and a protease inhibitor cocktail (Roche) was added. Then samples were centrifuged at 3000×g for 10 min in order to remove residual cells. The proteins from each sample were precipitated with 10% trichloroacetic acid after addition of one fourth volume of trichloroacetic acid at 50% (w/vol). The solutions were incubated for 30 min on ice. The samples were centrifuged for 30 min at 14,600 *g* at 4°C. The resulting pellets were dissolved with 30 μl of 2X LDS (Invitrogen) and then heated at 99°C for 5 min. Proteins were loaded on a 4–12% Tris-Bis NuPAGE gel (Invitrogen) for a 3-mm short migration. The bands containing the whole secretome were excised from the polyacrylamide gel and proteolysed in-gel with trypsin using the ProteasMax protocol (Promega, Charbonnières, France) as described previously [46]. The six resulting peptide mixtures were analyzed in duplicate by tandem mass spectrometry (MS/MS).

### Shotgun Proteomics of Secretome Samples

Nano-liquid chromatography-tandem MS (LC-MS/MS) experiments were performed using a LTQ-Orbitrap XL hybrid mass spectrometer (ThermoFisher) coupled to an UltiMate 3000 LC system (Dionex-LC Packings) in similar conditions as those previously described [47]. A volume of 10 μL of peptide mixture was loaded per sample. Peptides were resolved using a 120 min gradient from 5 to 50% solvent B (0.1% HCOOH/80% CH_3_CN), solvent A being 0.1% HCOOH. The activation type used was CID with a standard normalized collision energy set at 30. The lock mass option on the LTQ Orbitrap XL mass spectrometer was enabled in MS mode and the polydimethylcyclosiloxane ions was generated in the electrospray process from ambient air (protonated [(CH_3_)_2_SiO)]6 with m/z at 445.12002 uma) were used for internal recalibration in real time. Peak lists were generated with the extract_msn.exe data import filter of the Mascot Daemon software (version 2.3.3, Matrix Science) with the same options as previously reported (Hartmann and Armengo, in press). The MS/MS spectra were searched with MASCOT against a home-made database consisting of *Danio rerio* proteins (NCBI RefSeq database, release 130513) and 23 *Bos taurus* proteins previously identified as the main contaminants arising from fetal calf serum found in the cell culture medium. The database resulted in 27,367 entries totaling 14,426,496 amino acids. Standard MASCOT search parameters were applied: tryptic peptides with a maximum of two miss-cleavages, mass tolerances of 5 ppm on the parent ion and 0.5 Da on the MS/MS, fixed modification for carboxyamidomethylated Cys and variable modification for oxidized Met. MASCOT results were parsed using the IRMa 1.28.0 software [48] on the basis of a peptide p value below 0.05. A protein was considered valid when at least two different peptides were detected. The false-positive rate for protein identification was estimated using the corresponding reverse decoy database and these parameters as below 0.2%. Label-free protein quantitation by spectral counts was performed as previously described (Hartmann et al; 2013, in press). Cellular function and localization of identified proteins were predicted with the Ingenuity Pathway Analysis program (Ingenuity Systems). Student’s T-test was applied on spectral counts to assess differences between groups using SigmaStat (Systat software). When the normality test or the equal variance test failed, a Mann-Whitney rank sum test was performed.

### Statistical Analyses

The occurrence of gamma-H2AX (γ-H2AX) in each condition was compared to each other with analysis of variance (ANOVA) using Tukey’s all-pair comparisons test. For global methylation analysis, each methylation status was compared to each other. Normality assumption was checked by Shapiro test and a Mann-Whitney U-Test was used.

## Results

### 1 Chronic Irradiation Leads to DNA Damage Accumulation on Zebrafish Cells

**Figure 2:**
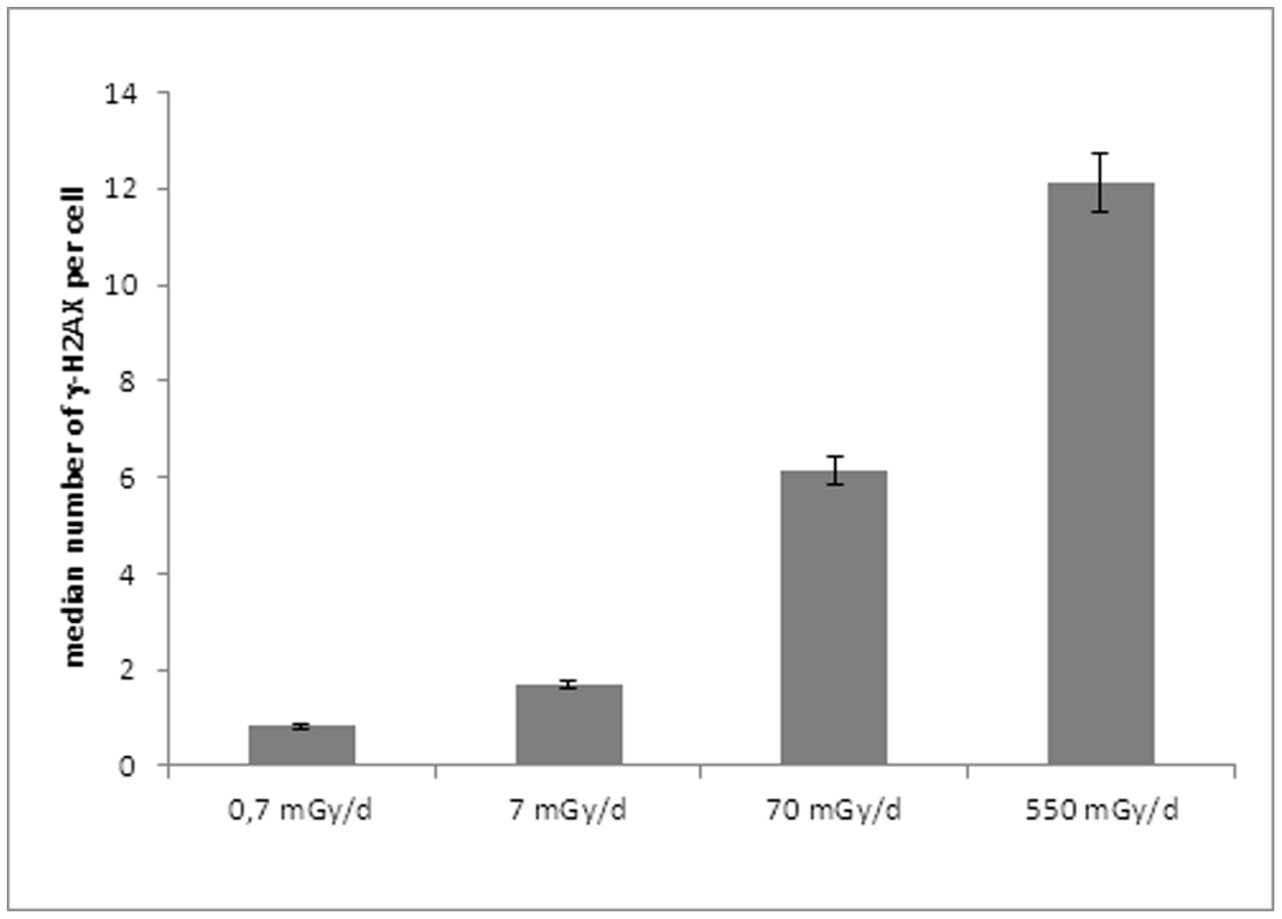
Assessment by H2AX immunostaining of DNA double strand breaks in chronic 24-irradiated ZF4 cells.

DNA damage and repair were analyzed on ZF4 cells after 24 h of exposure to gamma rays at 0.7, 7, 70 and 550 mGy/d (Figure 1). As a first step, we examined the occurrence of γ-H2AX foci on gamma irradiated ZF4 cells. This exposure led to the production of significantly higher numbers of γ-H2AX foci from a dose rate of 70 mGy/d compared to the background values in control cells (13.6±0.3 versus 1±1 γ-H2AX foci per cell, for cells exposed to 550 mGy/d and the control, respectively) (Figure 2). These results suggest the production of a significant number of persistent DSB after 24 h of chronic irradiation implying that the induced DSB were not easily reparable. Some similar results were previously shown with higher doses [49].

### 2 Induction of γ-H2AX Foci in Targeted and Bystander Cells at Very Low Doses of Gamma Irradiation

**Figure 3:**
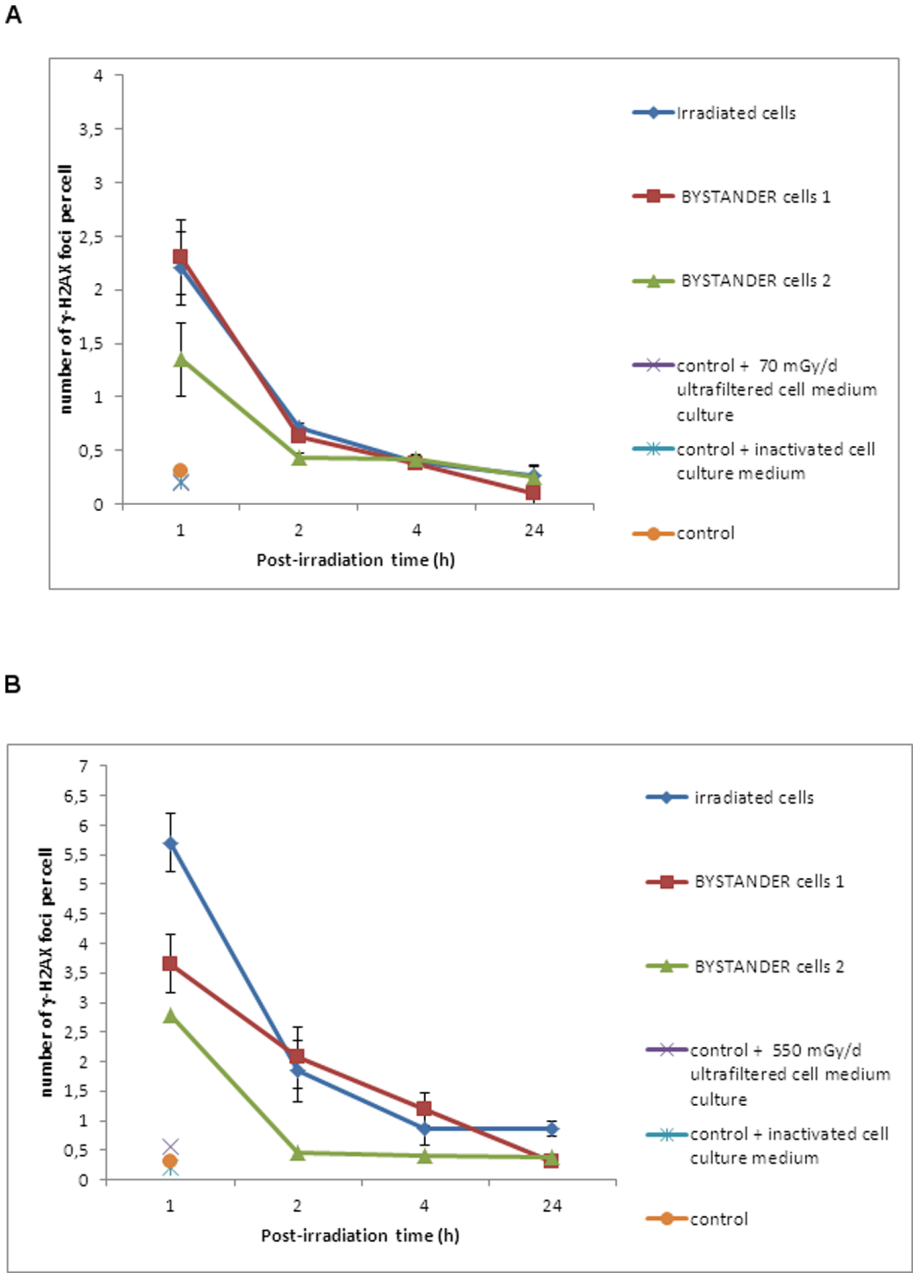
Exposure of ZF4 cells to gamma irradiation induces bystander responses in non-irradiated cells. A. Kinetics of γ-H2AX foci disappearance on 70 mGy/d gamma-irradiated ZF4 cells, respectively. B. Kinetics of γ-H2AX foci disappearance on 550 mGy/d gamma-irradiated ZF4 cells, respectively. Each data plot represents the mean+/−SE (n = 6) of at least 3 independent experiments.

To investigate the bystander effect and the response to DNA damages, ZF4 cells were irradiated for 4 hours at two different dose rates. Following irradiation, the medium from irradiated cells (bystander cells 1) or the irradiated cells were placed near non-irradiated cells (bystander cells 2) during 1 hour (see Figure 1 for detailed experimental set up). We then examined the presence of DNA double strand breaks at 1, 2, 4 and 24 hours post-irradiation via the detection of γ-H2AX foci in irradiated and bystander cells. Cells were treated with dose rates of either 70 mGy/d or 550 mGy/d for 4 hours, resulting in a total dose of 12 mGy (Figure 3A) or 92 mGy (Figure 3B) respectively. For both doses, the number of γ-H2AX foci was higher in irradiated cells than in bystander cells. An average of 2.3 foci (Figure 3A) or 5.8 foci (Figure 3B) was detected in the nucleus of irradiated cells 1 h after irradiation. For the lower dose (12 mGy, at the dose rate of 70 mGy/d), cells to which the culture medium from irradiated cells was transferred (bystander cells 1) presented a higher number of γ-H2AX foci 1 hour post-irradiation relative to cells that were co-cultured with irradiated cells (bystander cells 2) with an average of 2.2 vs 1.3 foci, respectively (figure 3A). This implies that a soluble factor contained in the culture medium of irradiated cells is responsible of the appearance of DNA double strand breaks in non-irradiated cells. Similarly, for the higher dose (92 mGy, at the dose rate of 550 mGy/d), the number of foci in bystander cells 1 (culture medium transferred) is higher than in bystander cells 2 (cells transferred), with an average of 3.6 vs 2.7 foci of γ-H2AX, respectively (Figure 3B).

**Figure 4:**
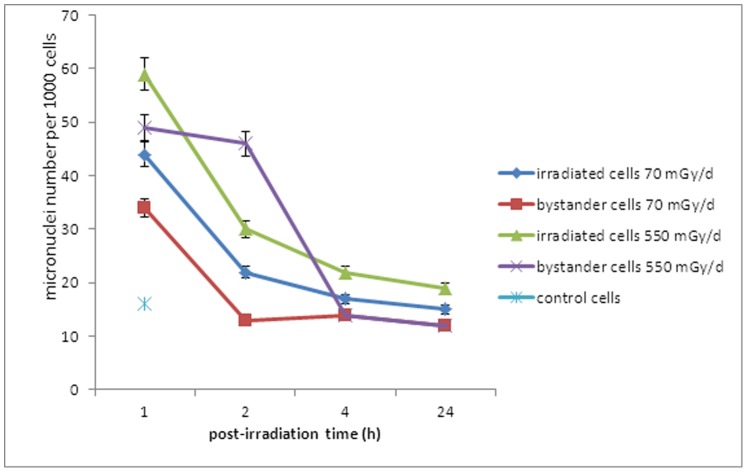
Exposure of ZF4 cells to gamma irradiation induces micronuclei in bystander cells. Micronuclei number was assessed on 1,000 cells stained with DAPI.

A significant part of the biomass of irradiated and bystander cells showed micronuclei supporting the formation of DSB during the gamma rays exposure. Bystander cells 2 exposed to cells irradiated at either 70 mGy/d or 550 mGy/d showed an elevated number of micronuclei per cell compared to control cells. Furthermore, bystander cells at 550 mGy/d presented numerous micronuclei relative to irradiated cells at 2 h post-irradiation, after which, a large decrease was noted (Figure 4).

These results showed that non-irradiated cells can present DNA double strand breaks when in contact with the culture medium of irradiated cells. It is also worth noting that these breaks are repaired within 24 hours (figures 3A and 3B). This implies that at these low doses the bystander effect observed does not impact the mechanisms of DNA double strand break repair in both targeted and bystander cells. The bystander effect observed here may be due to either a substance present in the sample and modified upon irradiation or a biological molecule such as a protein that is produced and secreted by the irradiated cells. This is theorized because the same type of results is obtained by transferring the culture medium of irradiated cells on non-irradiated cells.

### 3 Gamma Irradiation Affects Global Methylation in Both Irradiated and Bystander Cells

**Figure 5:**
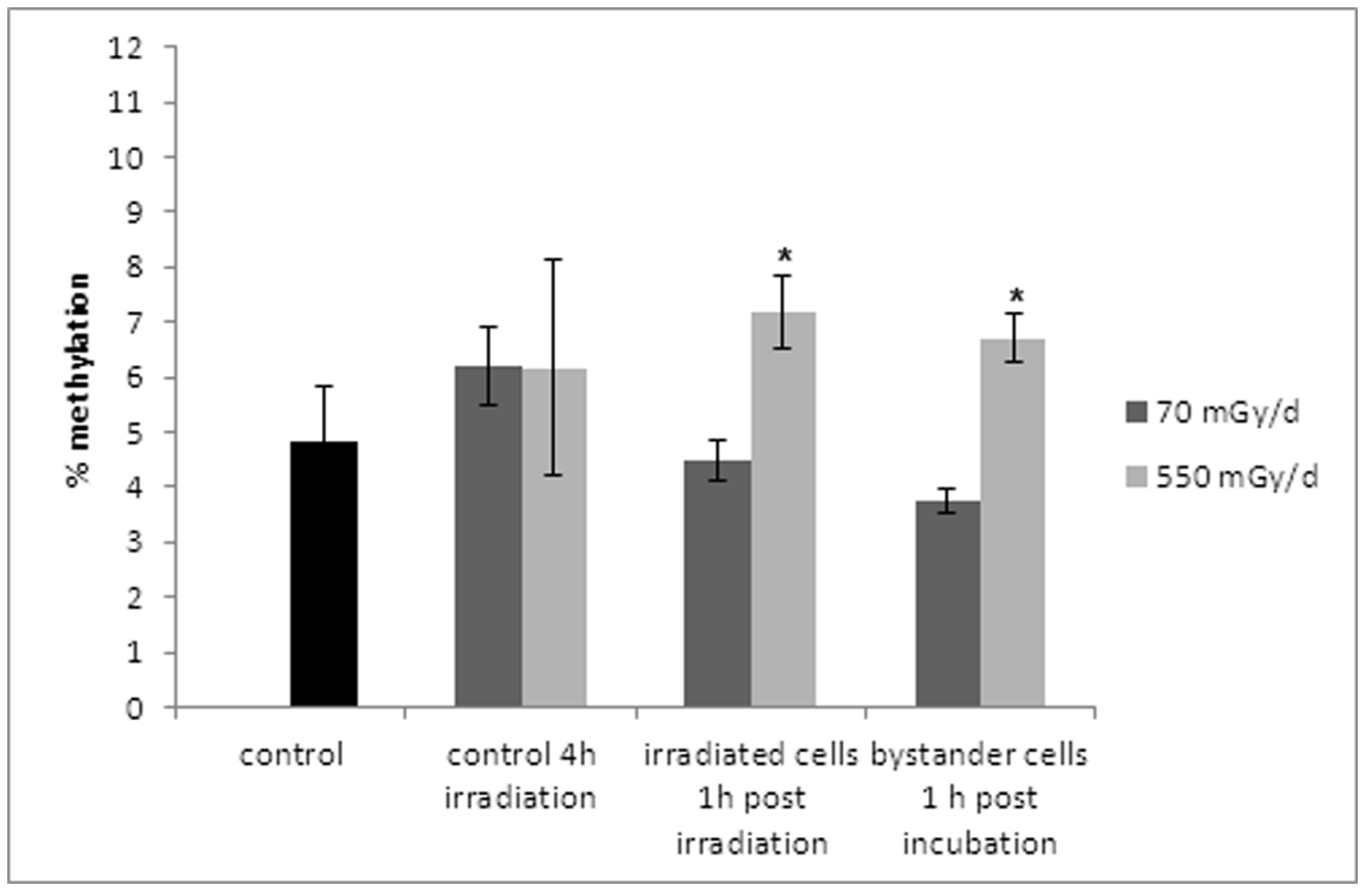
Gamma irradiation affects global methylation in irradiation and bystander cells. Methylation was measured by HPLC-MS. 3 μg of total DNA from irradiated and bystander cells were used. *p-value <0.5.

Figure 5 shows the level of DNA methylation from control, irradiated cells and bystander cells measured by mass spectrometry. An increase of global methylation of ZF4 cells was observed in irradiated cells and bystander cells compared to control cells. This increase was statistically significant (p<0.05) for the higher dose-rate (550 mGy/d) in both irradiated and bystander cells. Increased DNA methylation levels remained until 1 h post-irradiation (Figure 5), suggesting that epigenetic effects are concomitant with the appearance of DNA damages in bystander cells.

### 4 Early bystander Effect Leads to the Radioprotection of Non-irradiated Cells

**Figure 6:**
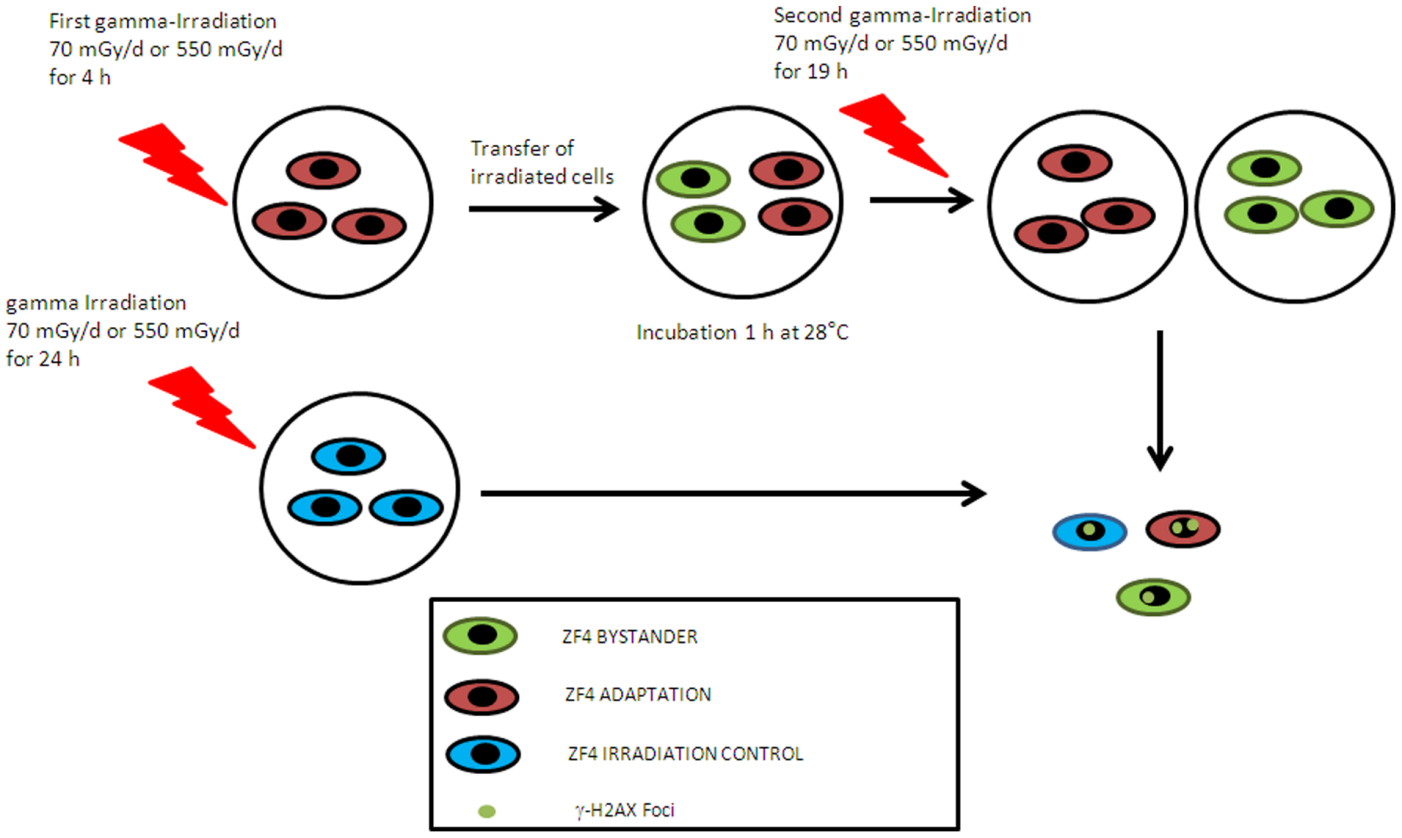
Experimental set up for radioadaptation experiments.

Some authors recently showed in alpha irradiated and non-irradiated zebrafish embryos, that the stress communicated between unirradiated zebrafish embryos and irradiated embryos sharing the same medium will help “rescue” the irradiated embryos. The strength of the rescue effect depends on the number of rescuing unirradiated bystander embryos [33], [34], [35]. Based on these studies, radioadaptation experiments were done on embryonic ZF4 cells (see Figure 6 for experimental set up). First, ZF4 cells were pre-irradiated for 4 hours at two different dose-rates. Then, these irradiated cells (called adaptation cells) were placed near non- irradiated cells (called bystander cells) for 1 hour. This incubation was followed by a second irradiation of adaptation and bystander cells for 19 hours. The number of γ-H2AX foci was assessed after the 24 hour-exposure time.

**Figure 7:**
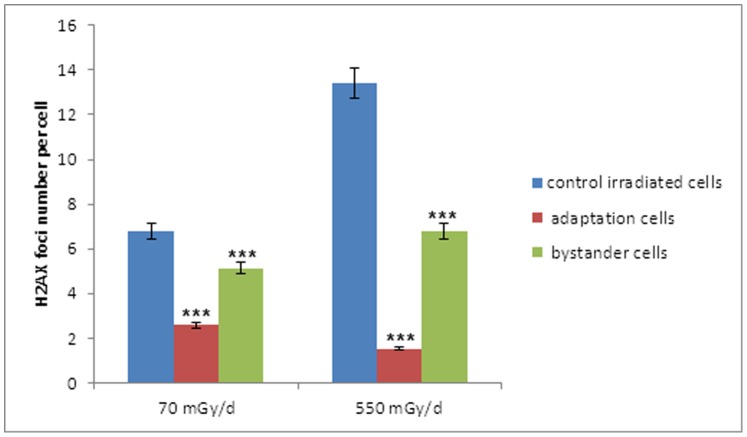
Early bystander effect leads to the radioprotection of non-irradiated cells. Pre-irradiated cells (called adaptation cells) were placed near non-irradiated cells (called bystander cells) for 1 hour. This incubation was followed by a second irradiation of adaptation and bystander cells for 19 hours. Assessment of γ-H2AX foci number was done on adaptation, bystander and control ZF4 cells 24 h after irradiation with gamma rays at a dose rate of either 70 mGy/d and 550 mGy/d. The difference in the formation of γ-H2AX foci was significant (***p-value <0.001, Student Test).

We observed that adaptation cells which are then irradiated for a second time contained significantly less foci for the higher dose than control cells that were continuously irradiated for 24 h with gamma rays (approximately 1.7 foci per cell, p<0.001) (Figure 5). We showed that bystander cells that have been in contact with adapted cells and were then irradiated for 19 h present less γ-H2AX foci than cells irradiated alone only during 24 hours (6.4 foci vs 13 foci per cell; p<0.001) (Figure 7). This radioadaptation effect was significantly more pronounced at the highest doses (p<0.001, Student test) (figure 7).

To assess whether the factor responsible for DNA damage in bystander cells was a protein or a low molecular weight metabolite, we applied the culture medium of irradiated cells to the non-irradiated bystander cells either i) ultrafiltration with a low molecular weight cut-off (3 kDa), or ii) denaturation by heating for 10 min at 100°C (see Figure 1). In both cases, no DNA breaks were observed and the number of γ-H2AX foci in the bystander cells was found to be similar to control non-irradiated cells (Figures 3A and 3B). This result suggests that the factor responsible of DNA damage in ZF4 cells has a molecular weight higher than 3 kDa and is inactivated by heating, pointing to a possible protein nature of the bystander mediator. With the aim of identifying the factor acting in early bystander effects and responsible of the radioadaptation of ZF4 cells, we studied the secretome of irradiated cells.

### 5 Early Bystander Effect is Not Directly Linked to the Secretion of a Protein by Irradiated Cells

We performed an extensive comparative study of the ZF4 secretomes upon irradiation. For this, we collected the conditioned media from two biological replicates from each condition (cells irradiated for 4 h or control cells). Proteins present in the conditioned media were concentrated and then resolved by SDS-PAGE, and proteolyzed with trypsin. The resulting peptides were analysed twice by nanoLC–MS/MS (technical replicates). From the MS/MS spectra data set recorded over the 12 resulting nanoLC–MS/MS runs (71,743 MS/MS queries), a total of 22,211 MS/MS spectra could be assigned (*p*-value below 0.05). These are listed in Table S1. They correspond to 1,628 peptide sequences pointing at the presence of 127 proteins from zebra fish. Table S2 reports the list of validated proteins, their characteristics and their spectral count quantification in the different samples. We predicted the cellular localization of these proteins using the Ingenuity Pathway Analysis (IPA) program. A total of 106 proteins were detected at least twice in the exoproteome of ZF4 control cells (un-irradiated) (Table S2, underlined in bold). A relatively low number of proteins present in the exoproteome of control cells are predicted to be extracellular (7%), indicating a low level of secretion of ZF4 cells after 4 h incubation while 56% are predicted to be cytoplasmic. That a relatively high number of intracellular proteins are found in ZF4 secretome was expected as this has been systematically observed in all previously published exoproteome studies [50]. Even if cell viability is very high (higher than 90%), these intracellular proteins are detected due to the high sensitivity of the mass spectrometer used here. Definitively, the number of secreted proteins observed for ZF4 cells in this condition is low compared to previous studies carried out with human cell lines [47]. This may be primarily due to the short incubation time, *i.e.* 4 h versus 24 h in most secretome studies. We can thus conclude that, after 4 h of incubation, the number of proteins secreted by ZF4 cells is moderate (Table S2, secreted proteins in bold).

When merging the exoproteome lists from irradiated and control cells, a total of 127 proteins were identified (Table S2), among which 10 were predicted as extracellular. No significant difference in their detection was evidenced between the samples on the basis of their normalized spectral counts (fold change of at least 2 and *p*-value below 0.05) (Table S3). A 2.7 fold increase was observed at 750 mGy for the complement component 7, but this was observed only for one of the two biological replicates. This indicates that a stress consisting of 4 hours of low dose of ionizing radiation does not induce major changes in secretion of a specific protein.

Oxydation of methionine could be a post-translational modification of proteins induced by ionising radiation. Thus, we analyzed whether the level of oxydized methionines was different among the samples (Figure S1). We directly extracted the number of this post-translational modification as seen from the list of peptides detected by tandem mass spectrometry (Table S1). A slight increase was observed in the irradiated samples compared to the controls: 2.7 and 2.9% vs 2.3, but this was not statistically significant (p-value >0.05). In the experimental conditions assayed here, the bystander effect is probably not due to the secretion of specific proteins or the oxidation of these secreted proteins although a slight increase of oxidation was noted (Figure S1).

## Discussion

The present study contributes to the understanding of early cellular and molecular mechanisms responsible for both the bystander effect and radioadaptation in embryonic ZF4 cells through quantification of DNA double strand breaks. While the bystander effect was intensively studied in mammalian cells, little information is available at the cellular level for the fish models [51]. Specifically, no one has addressed the occurrence of DSBs in irradiated and bystander fish cells after low gamma irradiation exposure <0.1 Gy. For this reason, we performed an experiment where ZF4 cells were irradiated in a range of doses between 0.01 and 0.1 Gy (*i.e.* 12 and 92 mGy) within an irradiation time of 4 h. Furthermore, the studied dose rates in chronic irradiation experiments were chosen around the existing benchmark level of 10 mGy/d recommended by the International Atomic Energy Agency (1992).

We showed that non irradiated cells incubated with irradiated cells (bystander cells 2) or with culture medium from cells irradiated (bystander cells 1) can present DNA double strand breaks (Figure 3). DNA damage observed in bystander cells 1 was higher than those observed in bystander cells 2 in a dose-dependent manner (Figure 3A and 3B) which implies that the factor responsible for this type of DNA damage is release by irradiated cells into the culture medium. It has been shown that bystander cells show a DNA damage response which is distinct from cells that are directly irradiated [52]. Indeed, we previously demonstrated that chronic irradiation clearly leads to the production of residual DSBs for dose rates >0.1 Gy/d and that this number of residual DSBs are due to an impairment of the non-homologous end joining repair pathway [49]. Here, bystander cells present the same kinetic of DNA repair as cells that were irradiated for 4 h. Furthermore, no residual DSBs were observed 24 h post-irradiation (Figure 3A and B) which implies that a chronic irradiation of 4 h does not disturb DSB repair mechanisms. Indeed, it has been shown that bystander-induced γ-H2AX foci colocalized with DNA damage checkpoint signaling and repair factors such ATM, MRE11, and RAD50 [16] which implies a functional DNA damage repair pathway.

It is now well established that embryo tissues are sensitive to radiation and may have apoptotic or other cellular death machineries to remove damaged DNA, such as DNA with DSBs, as has been observed in species such as zebrafish [42], [49]. Furthermore, some authors showed gamma radiation-induced reduction in bystander cell survival by the way of apoptosis [6], [53]. A high number of micronuclei were observed in cells irradiated at 70 mGy/d and 550 mGy/d and the same kinetic was observed for both conditions (Figure 3). Bystander cells at 550 mGy/d presented numerous micronuclei compared to irradiated cells at 2 h post-irradiation. This increase is concomitant with a higher number of DSBs in bystander cells 1 than in irradiated cells at 2 h post –irradiation (average of 2.2 foci vs 1.9 foci of γ-H2AX respectively; Figure 3B). This phenomenon was not observed for irradiated and bystander cells at 70 mGy/d (figure 3A). The presence of higher numbers of micronuclei suggests that from 1 to 24 h post-irradiation the major death mode of bystander cells seems to be a mitotic catastrophe, a form of cell death that results from abnormal mitosis and leads to the formation of interphase cells with multiple micronuclei (Figure 4). We also observed a hypermethylation of the DNA nucleic acid molecules in irradiated and bystander ZF4 cells for the higher dose, at 1 h post-irradiation, suggesting that epigenetic effects are concomitant with the appearance of DNA damages in bystander cells (Figure 5). Some studies showed that bystander exposure lead to changes in DNA methylation such as hypomethylation in rodents [54], [55] or hypermethylation observed in Chernobyl pines [56]. Because the exact mechanism for the induction of DNA methylation changes in irradiated tissue is currently unknown, we can speculate that the methylation changes are involved in mechanisms leading to bystander effect in non-irradiated cells.

Sawant and collaborators showed that bystander effect can be elicited in the same experimental system together with adaptative response [21], [50]. Furthermore, it has been shown that radiation-induced bystander effects may play a role in radioadaptive responses [32], [51]. *In vivo* studies in zebrafish embryos irradiated with alpha rays showed that radioadaptative response assessed by the number of apoptotic signals can occur in unirradiated embryos sharing the same medium than irradiated embryos [33]. Furthermore, Choi and collaborators showed zebrafish embryos irradiated with alpha rays can be rescued by unirradiated bystander zebrafish embryos [34], but the authors did not perform a challenging dose on irradiated and unirradiated embryos like in previous study [33].

We demonstrated that pre-irradiated embryonic ZF4 cells (called adaptation cells) contained significantly less foci than control cells irradiated for 24 h (Figure 5). These adapted cells may release a “protective factor” or “an inducer of protection mechanism” against DNA ionizing radiations effects as bystander cells that also protects bystander cells that have been in contact with adapted cells. Both bystander cells and adapted cells present less γ-H2AX foci when irradiated after 19 hours compared to control cells that were irradiated for 24 h (Figures 6 and 7). There was thus a “rescue” effect by cells that have not undergone pre-irradiation. The radioadaptation and the rescue effect appear to be dose-dependent as they are significantly more pronounced at high doses (figure 7). Similar phenomenon was observed in three fish cell lines for higher doses of gamma irradiation [51]. To identify the factor responsible for these early bystander effects and the radioadaptation of ZF4 cells, the exoproteome of ZF4 irradiated cells was studied with a high throughput shotgun proteomic approach. The so-called “exoproteome” comprise proteins from cellular secretion, other protein export mechanisms or cell lysis [50]. It has been shown that these proteins reflect the physiological state of the cells in a given condition and are indicators of how living systems interact with their environments [50]. In our study, after 4 h of irradiation, a relatively low number of proteins present in the exoproteome of control cells are predicted to be extracellular (7%), indicating a relatively low level of secretion of ZF4 cells while 56% are predicted to be cytoplasmic (Table S2). That a relatively high number of intracellular proteins are found in ZF4 exoproteome was expected as this has been systematically observed in all previously published exoproteome studies. Even if the results obtained indicated that 4 h of low dose of ionizing radiation does not induce a statistically significant change in the secretion of a given protein, we noted a 2.7 fold increase at 550 mGy/d of the complement component 7 (Table S3). These changes could indicate that higher secretion by ZF4 cells may occur for longer incubations.

Various studies identified TGF-beta1 [1], [52], [57], [15] as soluble factor acting in bystander effects signalling. These studies were performed with higher doses >0.01 Gy to 4 Gy and/or longer time of incubation and growth post-irradiation. In our study, the low dose-rate (0.07 and 0.55 Gy/d) applied for a short period of time (4 hours) could explain the low protein secretion of irradiated cells, which implies that in ZF4 cells the “early bystander effect” described can be due to another soluble factor rather than a secreted protein. Interestingly, a slight increase in the oxidation of the methionine residues in proteins from the exoproteome was observed in the irradiated samples compared to the controls. While this result is not statistically significant, it indicates a trend and we cannot exclude that factors other than oxidation of secreted proteins is involved in the “early bystander effect” observed in zebrafish cells. Indeed, this phenomenom is probably due to a combination of different factors. In human cells, some studies have shown that early events of bystander effect can be due to the generation of ROS but in all studies the doses applied were much higher than our study [6], [22], [57]. Further analyzes of the early events leading to a bystander effect in zebrafish are needed. Due to the close relationships between bystander and radioadaptation effects, what happens in the first hours post-irradiation in low doses irradiated cells should be investigated as this would help to better understand the adaptative resistance to ionizing radiations. The results of the present study provide new clues of zebrafish radiosensitivity and demonstrate that chronic low doses of gamma irradiation can induce bystander effect and radioadaptation in ZF4 cells. Further studies will be necessary to investigate the predictive biomarkers of a bystander effect. This could have important implications for environmental biomonitoring in relation to ionizing radiation exposure.

## Supporting Information

Figure S1 Level of oxydized methionines.
(DOC)

Table S1 List of redundant peptides obtained from the twelve replicates of control and irradiated samples.
(XLSX)

Table S2 List of MS/MS-identified proteins from Zebrafish.
(XLSX)

Table S3 List of extracellular proteins (IPA analysis).
(XLSX)
