# Supplementary material for: Low Doses of Gamma-Irradiation Induce an Early Bystander Effect in Zebrafish Cells Which Is Sufficient to Radioprotect Cells
Source: PLoS One. 2014 Mar 25;9(3):e92974. doi: 10.1371/journal.pone.0092974 (PMC3965492; doi:10.1371/journal.pone.0092974)
Supplement: Supplementary file 1 [file pone.0092974.s001.doc]

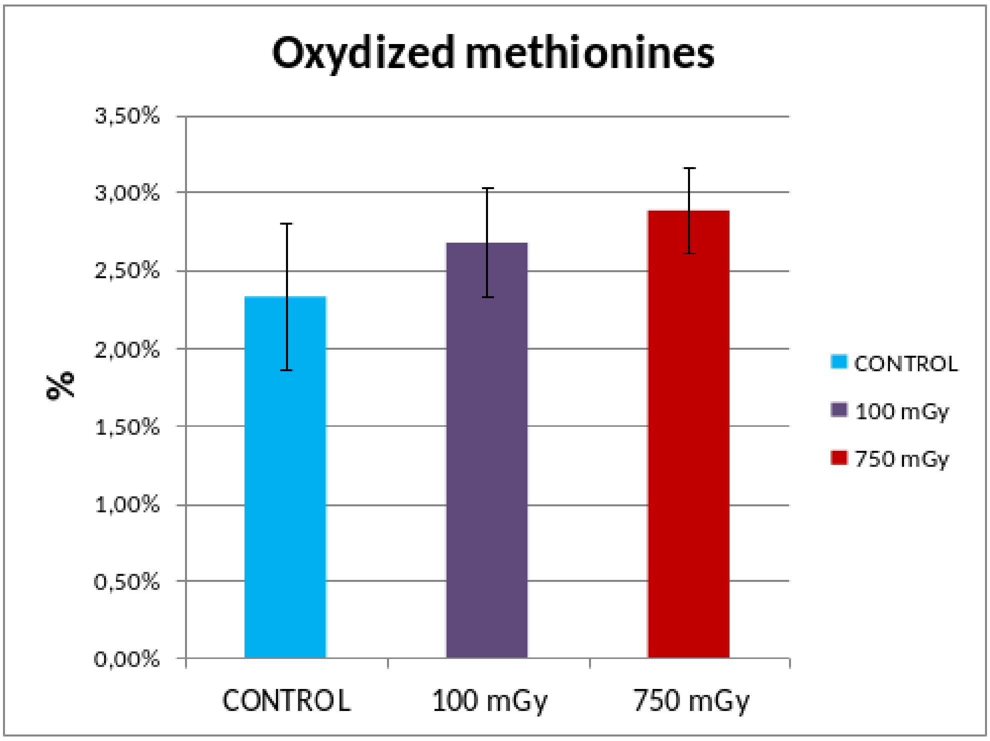
Oxydized methionines
%
3,50%
3,00%
2,50%
2,00%
1,50%
1,00%
0,50%
0,00%
CONTROL
100 mGy
750 mGy
CONTROL
100 mGy
750 mGy
